# The Specific Bile Acid Profile of Shock: A Hypothesis Generating Appraisal of the Literature

**DOI:** 10.3390/jcm9123844

**Published:** 2020-11-26

**Authors:** Lars-Olav Harnisch, Onnen Moerer

**Affiliations:** Department of Anesthesiology, University of Göttingen, 37075 Göttingen, Germany; omoerer@med.uni-goettingen.de

**Keywords:** bile acids, bile acid profile, critical illness, shock-pattern, deoxycholic acid

## Abstract

Background: Bile acid synthesis and regulation of metabolism are tightly regulated. In critical illness, these regulations are impaired. Consequently, the physiologic bile acid pattern in serum becomes disturbed and a disease-specific bile acid profile seems to become evident. Methods: A literature review was performed and trials reporting the broken-down bile acid pattern were condensed with regard to percent differences in bile acid profiles of defined diseases compared to a human control. Results: Ten articles were identified. Most of the studied bile acid profiles differ statistically significant between disease states, furthermore, neither of the reported disease entities show the same broken-down pattern of individual bile acids. Deoxycholic acid (DCA) was found to be decreased in almost all diseases, except for the two shock-states investigated (cardiogenic shock, septic shock) where it was elevated by about 100% compared to the control. Moreover, the pattern of both examined shock-states are very similar, rendering a specific shock-pattern possible, that we argue could eventually maintain or even worsen the pathological state. Conclusion: The specific broken-down bile acid profile of defined diseases might aid in gaining insight into the body’s adaptive reaction and the differential diagnosis, as well as in the therapy of disease states in the early course of the disease.

## 1. Introduction

Bile acids (Table 1) are amphiphilic molecules that are produced by the liver and secreted into the intestine where they solubilize lipids to aid their absorption; in the ileum, 95% of bile acids are actively reabsorbed, returned to the liver, and recycled (enterohepatic circulation).

However, bile acids do not only act as emulsifying agents for lipids in the intestine to aid their absorption. They also have an important regulatory impact on a large variety of organs and their functions, acting on them via transmembrane and intracellular receptors [1,2,3,4]. These effects are detectable, although the serum bile acid concentration is usually low due to a highly effective reuptake system in the gut [5].

Bile acid synthesis and the regulation of their metabolism are tightly controlled. Physiologically, primary bile acids and their conjugation products make up the main share of serum bile acids. In situations of either direct, such as obstruction of the bile duct, or indirect injury, such as critical illness, the regulation of bile acid metabolism was found to be impaired [6]. Consequently, the physiologic bile acid profile in serum becomes disturbed due to a variety of intracellular mechanisms and even on a translational basis [1,2,7]. This altered serum bile acid profile is likely to exert a variety of effects—because of the broad influence bile acids have within the human body—that are different from the physiologic state.

Shock states represent the most severe form of altered body functioning and they are associated with a severely increased mortality rate [8,9]. The different forms of shock have been defined according to their source (hypovolemic, distributive, cardiogenic, obstructive) [10]. The similarity of all shock states is that they result in an insufficient perfusion of vital organs that ends in tissue-hypoxia as a measure of discrepancy between oxygen supply and oxygen consumption [11].

To reverse the state of shock, the human organism relies on a uniform pattern of responses. One of these relatively uniform responses is a generalized vasoconstriction to redistribute the blood, and with it, the oxygen to the brain and heart at the expense of, for example, the splanchnic organs [11], which could serve as a partial explanation for impaired liver function and increased bile acid amounts in serum in these states.

So far, there is scarce knowledge on the informative value of the bile acid pattern and the disease specific profile of individual broken-down bile acids. We hypothesized that the broken-down profile of bile acids is not uniform as in systemic inflammatory response syndrome (SIRS), but rather an active, disease-specific adaptive response mechanism.

## 2. Methods

We searched the Medline database (via PubMED) combining the following MeSH (Medical Subject Headings) terms: Bile acid and either critical care, intensive care, or shock. We limited the search to original articles in humans that were available in English or German, with no limitation made with regard to the date of publication. The database was searched twice, once in March and again in November 2020 to be as up-to-date as possible before publication. All found articles were individually scanned for eligibility; of the identified studies, articles were additionally discarded if they did not present separation into individual bile acids. In addition, comparison to a control group was required for positive selection. Finally, the selected articles were scanned for references on relevant studies with the same selection criteria as aforementioned. The two investigators (L.-O.H. and O.M.) reviewed the identified abstracts independently and agreed on the final selection for inclusion; full texts were available for all studies finally selected. The study was conducted on the basis of the Preferred Reporting Items for Systematic Review and Meta-analysis (PRISMA) guideline.

Data extracted were the broken-down bile acid pattern as completely as presented in the respective study as well as the total amount of bile acids. If more than one illness/pathologic condition was presented in the study, data were extracted separately. To account for differences in the methods used to quantify the individual bile acids, values of the pathologic condition were put in relation by calculating the percent of change from the respective control group.

Statistical analysis was conducted with regard to differences of individual bile acids between the disease states extracted from the found articles using a multifactorial ANOVA (analysis of variance). For statistical analysis, we used SPSS (International Business Machines Corporated (IBM), Armonk, NY, USA, Version 26.0), statistical significance was assumed at *p* < 0.05.

## 3. Results

A total of 317 articles were identified, of which 311 met exclusion criteria of some kind, an additional 4 articles were detected by scanning references, resulting in a total of ten papers for analysis. The second search performed eight months after the first search revealed no additional studies. Table 2 summarizes the identified studies, with regard to the presented individual bile acid profile of the respective diseases/pathologic condition. Of all ten articles reviewed, only a slim minority presents the complete human broken-down pattern of individual bile acids and their conjugation products. The remaining articles charted present only an incompletely broken-down bile acid profile decreasing strength of comparison. Furthermore, every disease-state was studied only once.

In all reported trials, the total amount of bile acids (TBA) was increased above the upper limit of normal, the difference between the pathologic conditions was statistically significant (*p* = 0.022). The vast majority of individual bile acids and conjugation products in all of the studies was increased (Table 2). In contrast, deoxycholic acid (DCA) was either decreased (nine studies) or not altered (one study) in all disease states, except for the only two shock-states investigated (cardiogenic shock, septic shock), where it was significantly increased by almost/exactly 100 percent, respectively.

Changes in individual bile acids between the pathological conditions investigated were statistically significant for TCA (*p* = 0.048), TCDCA (*p* = 0.011), GCDCA (*p* = 0.002), DCA (*p* = 0.026), TDCA (*p* = 0.005), TUDCA (*p* = 0.004), GUDCA (*p* = 0.017), LCA (*p* = 0.028), GLCA (*p* = 0.033), and the changes in the total amount of bile acids (TBA, *p* = 0.022) but not for CA (*p* = 0.120), GCA (*p* = 0.06), CDCA (*p* = 0.067), GDCA (*p* = 0.106), and UDCA (*p* = 0.105).

## 4. Discussion

In all the studies analyzed, the total amount of bile acids (TBA) was consistently increased (or not presented in one study), which is explained by a reaction according to the toxic-bile-hypothesis [21]. This general increase disqualifies the total amount of bile acids in serum as a biomarker to differentiate between disease states but might suit to estimate a liver involvement early on. However, it clearly shows that the liver and especially the bile are affected in all critically ill patients at a rather early point during the course of the disease, a finding that has been described before [22]. This finding is in strong contrast to perceived regular clinical practice where secondary liver injury is a late, often additionally found, and frequently final disease state following a (long) course of critical illness.

Differentiation into the individual bile acids reveals a diverse bile acid profile between the different disease states studied. The proposed explanatory mechanism for this altered broken-down bile acid pattern in general is an impaired efflux into the bile, which has been described in an ex vivo model [22]. Most bile acids are toxic to hepatocytes [23], consequently the efflux into the blood in cholestatic conditions via MRP3 and MRP4 (multidrug resistance protein) transporters on the basolateral membrane of the hepatocyte is promoted presumably as a self-defense mechanism [24,25,26,27]. An alternative explanation would be the downregulation of the sodium-dependent taurocholic cotransporting peptide (NTCP) and organic anion transporting peptide (OATP)1B1 which has also been described [28,29,30]; a combination of both mechanisms might also be conceivable.

It is noteworthy that in the presented studies, none of the disease entities revealed the same broken-down pattern of individual bile acids. Not even disease entities pathophysiologically closely related—like obstructive cholestasis and biliary obstruction—show the same pattern (Table 2). The fact that bile acid pattern of even closely related diseases differ, favors an active and specific adaptive mechanism of altered production of bile acids, much more than the theory of a sole passive organ damage with bile acids being a biomarker of organ damage to our mind.

The first two lines of Table 2 show a rather similar bile acid profile for general liver disease and drug-induced liver injury. In this study, samples were taken “from patients with a broad range of liver impairments” [12]. Since bile acid profiles seem to be disease-specific, they should not have been evaluated pooled together in a general group but rather stratified into disease specific subgroups. Furthermore, drug-induced liver injury is one of the most common causes of acute liver failure and ultimately a diagnosis of exclusion [31]. From this, it is very likely that at least some of the patients with a broad range of liver impairments mentioned in this study suffered (amongst others) from drug induced liver injury. This would explain the closely related but not alike bile acid pattern found in both studies.

However, while most of the changes from control are within the same range, CA, TDCA, and GDCA differ 3- to 7-fold between these two pathological states. These differences could prove to have an extraordinary value in the differential diagnosis of acute liver injury, therapeutic, and predictive merit included. Of course, this needs verification in further dedicated trials.

Shock states are a serious condition and associated with increased mortality. Although the underlying cause differs, the pathophysiologic mechanism of microcirculatory impairment is a common feature. The broken-down bile acid profiles of both examined shock states are very similar (last two lines of Table 2), differing from all other states, especially with regard to deoxycholic acid (DC) that was only found to be increased in shock states; this finding renders a specific shock-profile likely.

**Figure 1:**
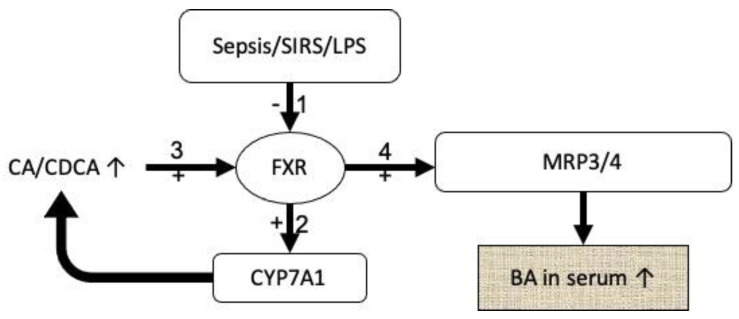
Possible mechanism of increased bile acids in serum during critical illness; symbols: - = inhibition, + = activation, ↑ = increase. Sepsis, the systemic inflammatory response syndrome (SIRS) as well as lipopolysaccharides (LPS) from the outer membrane of gram-negative bacteria have been shown to inhibit farnesoid-X receptor (FXR) expression. This reduced expression in turn induces the cytochrome-P 7A1 subtype (CYP7A1) which leads to an increased production in cholinic acid (CA) and chenodeoxycholic acid (CDCA). This increase activates the FXRs available that in turn activates and increases the number of MRP3 and MRP4 (multidrug resistance protein) transporters, finally reducing the concentration of toxic bile acids in the hepatocytes by exporting them into the serum where they can be measured elevated.

**Figure 2:**
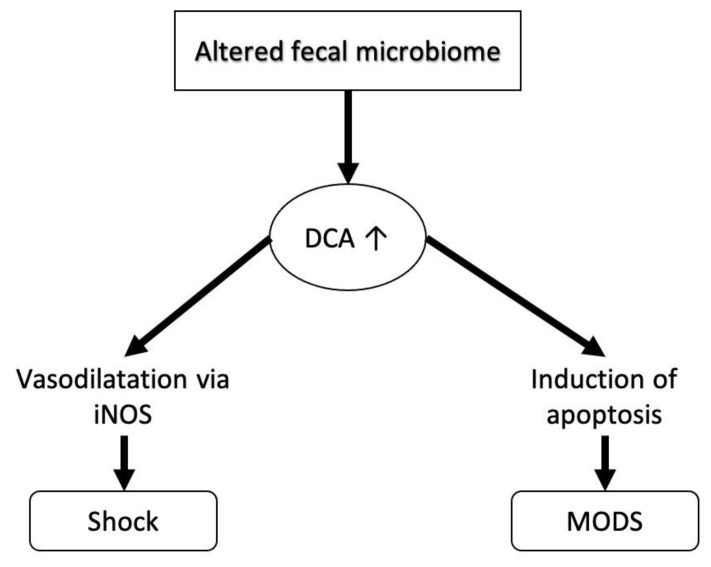
Likely mechanism and effects of increased (↑) deoxycholic acid (DCA) levels in serum during shock states. An altered fecal microbiome has been shown in recent studies. This altered microbiome leads to increased levels of deoxycholic acid in the intestine that are consecutively absorbed into the blood to be returned to but are not cleared by the liver and consequently are elevated in the serum. Known effects of deoxycholic acid include vasodilation mediated by the inducible nitrogen-oxydase (iNOS) and induction of apoptosis that can, in the extreme forms of shock lead to multi organ dysfunction syndrome (MODS).

We propose two possible mechanisms that might lead to this profile (Figure 1 and Figure 2).

With regard to raised bile acids in general, it has been shown already, that LPS (lipopolysaccharide, an endotoxin from the outer membrane of gram-negative bacteria) and SIRS (systemic inflammatory response syndrome) in general decrease expression of the nuclear farnesoid-X receptor (FXR) [6,32]. This reduction of FXR increases expression of CYP7A1 (cytochrome-P 7A1) that increases to the production of new bile acids [20], which in turn are excreted into the serum by the basolateral efflux-pumps MRP3 and MRP4 [21] and can thus be measured elevated. Since this increase in bile acids is most likely an active process rather than a passive reaction to an extreme state of illness, further basic research will need to elate on the beneficial effects of these alterations; some of which have already been described [13,33].

With regard to increased DCA-levels, an altered fecal microbiome has been found in critically ill patients already [14,15,16], while a link with increased disease-severity or mortality could not (yet) be established. However, since fecal bacteria play a pivotal role in the conversion of bile acids before reabsorption from the intestine, an altered microbiome most likely results in a changed serum bile acid profile [34]. Due to the elevated portion of DCA, its specific actions that otherwise will be overshadowed or countered by other mechanisms will come to the fore. The specific actions triggered by DCA include vasodilation [4] and induction of apoptosis [17], resulting in their extreme states in circulatory depression and multi organ dysfunction, both of which are found in shock-states.

From the thorough understanding of these mechanisms, possible therapeutic options might arise: Just recently it has been shown in an animal model that supplementation of an in a shock state decreased bile acid (TUDCA), exerts beneficial effects with regard to reduced apoptosis of hepatocytes [18]. In humans, fecal microbiota transplantation has been proposed as a promising option to treat septic conditions [19,20]. Furthermore, since conjugation of bile acids with taurine or glycine is dependent on the supply of the respective amino acid, this might offer another way of relatively easy influencing the composition of the bile acid pool.

Above that, because bile acids have been shown to be detectably increased very early during the course of a disease, it might also enable physicians to diagnose and treat shock states early on, possibly attenuating its length and severity with the appropriate therapeutic measures.

However, much more research has to be conducted within this field: There are many more disease states that are seen and treated in intensive/critical care units than those condensed here, like trauma, cardiac diseases, postoperative states, various forms of kidney injury, subgroups of septic conditions, acute respiratory distress syndrome (ARDS), or other shock states (hemorrhagic, anaphylactic). These entities have not yet been characterized with regard to their bile acid pattern. It will be interesting and necessary to see their specific profiles to understand the pathophysiology further and ideally also to generate therapeutic options.

Further trials within this field should necessarily present a complete breakdown into all individual bile acids and conjugation products that will enable researchers to define and straighten out disease-specific bile acid profiles.

This review is obviously limited by the inconsistent broken-down bile acid patterns presented in the included trials but also the limited number of trials and patients included.

## 5. Conclusions

Severe disease states and critical illnesses most likely have a specific bile acid pattern which—the likely active adaptive mechanism presumed—can aid in understanding their pathophysiology as well as the body’s reaction to these diseases. A specific shock-profile would not only aid in understanding the pathophysiology of shock further but could also facilitate supportive and therapeutic measures for its resolution. Even an earlier differential diagnosis is presumably imaginable. Further extensive research will be needed to confirm and further elate on these facts.

## Figures and Tables

**Table 1:** Human bile acids, their conjugation products and abbreviations used in this article.

Bile Acid	Abbreviation
Cholinic acid	CA
Taurocholic acid	TCA
Glycocholic acid	GCA
Chenodeoxycholic acid	CDCA
Taurochenodeoxycholic acid	TCDCA
Glycochenodeoxycholic acid	GCDCA
Ursodeoxycholic acid	UDC
Tauroursodeoxycholic acid	TUDCA
Glycoursodeoxycholic acid	GUDCA
Litocholic acid	LCA
Taurolitocholic acid	TLCA
Glycolitocholic acid	GLCA

**Table 2:** Individual bile acid pattern of the respective diseases/pathologic condition presented as percent change from the respective control group.

Disease Category	Disease State Investigated	CA	TCA ^#^	GCA	CDCA	TCDCA ^#^	GCDCA ^#^	DCA ^#^	TDCA ^#^	GDCA	UDCA	TUDCA ^#^	GUDCA ^#^	LCA ^#^	TLCA ^#^	GLCA ^#^	TBA ^#^
Liver	General liver disease [12]	+210 *	+9622 *	+2769 *	+141 *	+5225 *	+1279 *	−57 *	+593 *	+215 *	n/a	n/a	n/a	n/a	n/a	n/a	+969 *
	Drug induced liver injury [12]	+1343 *	+7856 *	+5159 *	+423 *	+5959 *	+2776 *	−46 *	+1519 *	+1192 *	n/a	n/a	n/a	n/a	n/a	n/a	+2192 *
	Viral hepatitis [13]	+33	+1331	+559	−26	+659	+522	−52	+250	+48	+20	+1100	+326	+135	+150	+229	+420
	Alcoholic liver disease [13]	+103	+1119	+653	+60	+599	+589	−70	+77	−7	+482 *	+2589	+1482 *	+114	+82	+103	+460
	Non-alcoholic fatty liver disease [13]	+192	+619	+426	+70	+265	+382	−25	+186	+61	+65	+511	+245	+236	+67	+300	+366
	Other liver diseases [13]	+28	+3	+1060	+54	+957	+513	−72	+191	+14	+14	+1584	+416	+73	+133	+227	+457
	Acutely decompensated chronic cirrhosis [14]	+25	n/a	+228 *	+102 *	+356 *	+136 *	−63	−100	−82	+33	0	+413 *	−14	−100	+67	+195
Biliary Tract	Biliary obstruction [15]	+5	+30,221 *	+24,030 *	−67 *	+13,692 *	+3599 *	−95 *	+375 *	+70	−85 *	+1398 *	n/a	+88 *	+50	+36	+5778 *
	Obstructive cholestasis [16]	0	+3 *	+2 *	−55	+2 *	+689 *	−90 *	+300 *	+375 *	0	n/a	n/a	0	n/a	n/a	n/a
	Severe cholestasis in pregnancy [17]	+134 *	+5411 *	+1053 *	−60	+210 *	+342 *	−26	+1082 *	+125 *	−37	+73 *	n/a	0	−25	0	+767 *
	Biliary tract disease [13]	+9	+4295 *	+1491	+70	+1723	+798	−88 *	+309	+14	−55 *	+1323	+276	+72	+193	+114	+709
Other	Hepatopulmonary syndrome [18]	+63 *	+645 *	+216 *	+856 *	+464 *	+210 *	−53	−100	−100	+25	−100	+75	−38	−33	−100	+210 *
	General critical illness [19]	+25	+2050 *	+8200 *	−10	+3800 *	+3300	0	0	0	0	0	0	0	0	n/a	+1010 *
Shock States	Cardiogenic shock [20]	0	+650 *	+1067 *	−10	+2600 *	+580 *	+100 *	+533 *	+2800 *	+300 *	+800 *	+200 *	+25 *	+600 *	n/a	+90 *
	Septic shock [20]	0	+1150 *	+1933 *	+120	+4900 *	+1820 *	+80 *	+333 *	+5500 *	+600 *	+1000 *	+350 *	0	+500 *	n/a	+590 *

Statistically significant differences from the respective control group are marked * (extracted from the original articles), statistical significances of individual bile acids between conditions are marked ^#^ at the respective bile acid in the heading of each column. Abbreviations: CA = cholinic acid, TCA = taurocholic acid, GCA = glycocholic acid, CDCA = chenodeoxycholic acid, TCDCA = taurochenodeoxycholic acid, GCDCA = glycochenodeoxycholic acid, DCA = deoxycholic acid, TDCA = taurodeoxycholic acid, GDCA = glycodeoxycholic acid, UDCA = ursodeoxycholic acid, TUDCA = tauroursodeoxycholic acid, GUDCA = glycoursodeoxycholic acid, LCA = litocholic acid, TLCA = taurolitocholic acid, GLCA = glycolitocholic acid, TBA = total bile acids, n/a = data not available from the original article.
